# Botulinum Toxin Therapy for Managing Sleep Bruxism: A Randomized and Placebo—Controlled Trial

**DOI:** 10.3390/toxins12030168

**Published:** 2020-03-09

**Authors:** Young Joo Shim, Hee Jin Lee, Keun Jeong Park, Hyung Tack Kim, Il Hee Hong, Seong Taek Kim

**Affiliations:** 1 Department of Oral Medicine, College of Dentistry, Wonkwang University, Daejeon 35233, Korea; gc21@wku.ac.kr; 2 Department of Orofacial Pain and Oral Medicine, Yonsei University Dental Hospital, Seoul 03722, Korea; hijin5500@gmail.com (H.J.L.); 68keunjeong@gmail.com (K.J.P.); 3 TMJ & Orofacial Pain Clinic, Los Angeles, CA 90006, USA; sontec-kim@daum.net; 4 Seoul Sleep Clinic, Seoul 06052, Korea; ilheehong2@gmail.com; 5 Department of Orofacial Pain and Oral Medicine, College of Dentistry, Yonsei University, Seoul 03722, Korea

**Keywords:** botulinum toxin, sleep bruxism, polysomnography

## Abstract

The purpose of this study is to evaluate the effects of botulinum toxin type A (BoNT-A) for managing sleep bruxism (SB) in a randomized, placebo-controlled trial. Thirty SB subjects were randomly assigned into two groups evenly. The placebo group received saline injections into each masseter muscle, and the treatment group received BoNT-A injections into each masseter muscle. Audio–video–polysomnographic recordings in the sleep laboratory were made before, at four weeks after, and at 12 weeks after injection. Sleep and SB parameters were scored according to the diagnostic and coding manual of American Academy of Sleep Medicine. The change of sleep and SB parameters were investigated using repeated measures analysis of variance (RM-ANOVA). Twenty-three subjects completed the study (placebo group 10, treatment group 13). None of the SB episode variables showed a significant time and group interaction (*p* > 0.05) except for electromyography (EMG) variables. The peak amplitude of EMG bursts during SB showed a significant time and group interaction (*p* = 0.001). The injection decreased the peak amplitude of EMG bursts during SB only in the treatment group for 12 weeks (*p* < 0.0001). A single BoNT-A injection cannot reduce the genesis of SB. However, it can be an effective management option for SB by reducing the intensity of the masseter muscle.

## 1. Introduction

In the *International Classification of Sleep Disorders,* 3rd edition [1], sleep bruxism (SB) is defined as stereotyped oromandibular activity during sleep characterized by teeth grinding and clenching. SB is common, with a prevalence of 7.4% of the adult population [2]. In dentistry, SB has been recognized as a risk factor related to teeth and dental prosthesis destruction, and pain in the masticatory structures [3,4]. Dental problems are associated with a significant amount of force during SB, averaging 66% of maximal clenching power [5].

There are treatment modalities for the management of SB, such as an occlusal splint, behavioral approaches, and pharmacological management. The occlusal splint is considered to be the first choice for protecting teeth and prostheses from damage. However, evidence that an occlusal splint reduces SB is lacking [6,7,8]. Botulinum toxin (BoNT) injection is widely used for SB management [9,10,11,12,13,14]. We showed that BoNT injection cannot control the genesis of rhythmic masticatory muscle activity (RMMA), but it can control the intense contractions of masticatory muscles during sleep in an observation study without a placebo group [15]. To clarify the difference between the efficacy (the effects on SB episodes) and effectiveness (the effects on the SB consequences) of BoNT for SB, well-designed study is needed [16].

In this study, extending the research of our previous study, we focused on the efficacy of BoNT-A for SB. We investigated the effects of intramuscular BoNT-A injection on SB using polysomnographic evaluation for 12 weeks in a randomized and placebo-controlled trial.

## 2. Results

**Figure 1:**
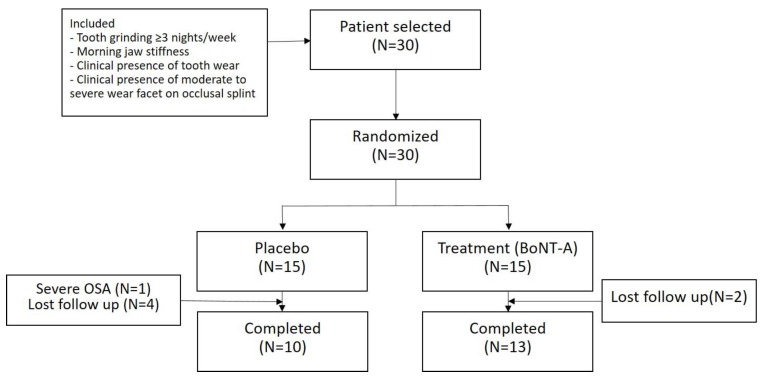
Study flowchart. BoNT-A, botulinum toxin type A; OSA, obstructive sleep apnea.

Among the 30 subjects, one was excluded because he showed severe obstructive sleep apnea on polysomnography (PSG) with an apnea–hypopnea index of 70.3, and six were unavailable for a follow-up PSG recording. Thus, 69 PSG recordings from the 23 subjects (10 from placebo group and 13 from the treatment group) were used for the analysis (Figure 1). The baseline characteristics of the subjects are listed in Table 1. There was no significant difference between the groups. The injection was well tolerated, and a significant adverse event related to the injection was not reported.

### 2.1. Sleep Variables

Data for the sleep variables are given in Table 2. All sleep variables showed no significant time and group interactions (repeated measures analysis of variance (RM-ANOVA), *p* > 0.05). Percentages of N2 and REM stages and sleep efficiency changed significantly in the placebo group during the 12 weeks. Total sleep time, percentage of N1 stage, and arousal index changed significantly in the treatment group during the 12 weeks.

### 2.2. Sleep Bruxism Episodes

Data for the parameters of SB episodes are given in Table 3. None of these parameters differed significantly between the groups at baseline (two-sample *t*-test, *p* > 0.05). None of the SB episode variables showed a significant time and group interaction (RM-ANOVA, *p* > 0.05) except for electromyography (EMG) variables. RMMA episode variables did not change significantly during the 12 weeks in either group except for the number of RMMA episodes per hour of sleep (RMMA episodes/h) in the placebo group (*p* = 0.036). For RMMA episodes/h variable, significant differences were found between baseline and 4 weeks (*p* = 0.011), and between 4 weeks and 12 weeks (*p* = 0.020) in the placebo group.

**Figure 2:**
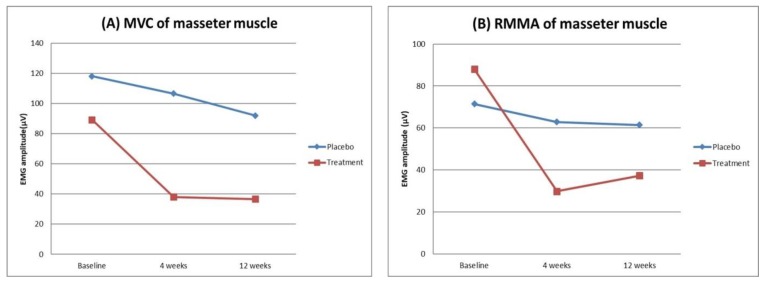
The change of amplitude of EMG burst in the masseter muscle during MVC (**A**) and RMMA (**B**). Graphs show significant time and group interactions. The amplitude of EMG burst in the masseter muscle during MVC and RMMA significantly decreased only in the treatment group for 12 weeks. MVC, maximal voluntary clenching; RMMA, rhythmic masticatory muscle activity.

In EMG variables, the peak amplitude of EMG burst in the masseter muscle during maximal voluntary clenching (MVC MA) and the peak amplitude of EMG burst in the masseter muscle during RMMA (RMMA MA) showed significant time and group interactions (RM-ANOVA, *p* = 0.044 for MVC MA, *p* = 0.001 for RMMA MA). MVC MA and RMMA MA significantly decreased only in the treatment group for 12 weeks (RM-ANOVA, *p* < 0.0001 for both MVC MA and RMMA MA). For these variables, a significant difference was found only between baseline and 4 weeks (paired *t*-test, *p* = 0.001 for MVC MA, *p* < 0.0001 for RMMA MA). Figure 2 shows the change of amplitude of EMG burst in the masseter muscle.

## 3. Discussion

This study showed that a single BoNT-A injection decreased the masseter muscle intensity during SB for 12 weeks by PSG evaluation in a randomized, double-blind, placebo-controlled trial. The findings of this study confirm that the effect of BoNT-A on SB reduces the intensity of the contractions in the injected muscles rather than reduces RMMA occurrence, and this effect was maintained for at least 12 weeks.

BoNT-A injections did not decrease the RMMA episodes/h. In addition, bursts/episode and mean episode duration did not differ before and after BoNT-A injection. These results are consistent with our previous study, in which we evaluated the effect of BoNT-A on SB using PSG for the first time in the literature and confirmed that BoNT-A did not affect the occurrence of RMMA [15]. In this study, we confirmed this again, in a randomized, double-blind, placebo-controlled study and for a longer period. These results are inconsistent with those of previous controlled trials, in which the number of bruxism events was favored in the BoNT-injection group [13,17]. One randomized, controlled 12-week study showed a decreased number of SB events [13]. That result should be interpreted with caution, because of the lack of PSG data and the small sample size. The other randomized, controlled 4- to 8-week study using PSG evaluation showed subjectively improved bruxism symptoms, fewer bruxism events, and less total bruxing time, but this was not evaluated by statistical analysis [17]. The discrepancy may be explained by the differences of the bruxism scoring methodology and study protocol. The investigators used a different bruxism scoring method, relatively large BoNT-A doses, and different injections sites (both masseter and temporalis muscles). Because application of BoNT-A is an off-label use in dentistry, there are no clinical protocols or standardization of dosage and preparations. Although PSG is the gold standard for SB diagnosis, it is not widely applied to clinical research and clinical fields because of its high cost, technical requirements, and need for expert examiners. These problems lead to inconsistency of study protocols, SB diagnostic tools, bruxism scoring methods, and results between studies. Well-designed, placebo-controlled studies with large sample on SB evaluated by PSG are needed to establish the efficacy of BoNT-A for SB.

SB is highly variable over time, with subjects showing no activity on some nights and intense activity on others. RMMA episodes/h changed significantly in the placebo group in this study, which shows the typically time-variant nature of SB. The previous study reported a mean coefficient of variation for RMMA episodes/h of about 25.3% [18]. In this study, there was a mean coefficient of variation for RMMA episodes/h of approximately 32.1% over three nights in the placebo group and 22.3% over three nights in the treatment group. Variability in SB should be taken into consideration when SB is being diagnosed or the intervention effect of SB management is being evaluated. For this time-variant nature of SB, one study suggested that PSG SB cut-off bands around SB cut-off points (4 episodes/h and 25 bursts/h) might be useful for the recognition of SB [19].

As expected and as all the previous studies have shown, the intensity of masseter muscle significantly decreased after BoNT-A injection, especially in 4 weeks. Recently, SB is not considered to be a movement disorder or sleep disorder in otherwise healthy individuals in international consensus panel [20]. In healthy individuals, SB would not be a disorder, but rather a behavior that can be a risk factor for certain negative clinical consequences, such as masticatory muscle pain or TMJ pain, mechanical tooth wear, and prosthodontic complications. RMMA is a physiological activity of jaw muscles during sleep and not harmful itself [21]. RMMA is regarded as a final consequence of micro-arousal [22,23]. SB is an extreme manifestation of this physiological RMMA during sleep whereby certain factors increase its occurrence [24,25]. Changing point of view and uncovering the pathophysiology of SB, we need not control the RMMAs per se in healthy individuals. Instead, we need to control the intensity of SB events. Conventionally, clinicians prescribe an occlusal splint on SB to prevent the damage. However, there was no consensus about the effect of occlusal splints on the intensity of the masseter muscle during SB. The previous studies showed that the intensity of masseter muscle during SB was decreased, increased, or not changed when wearing occlusal splint [6,7,8]. The effect of BoNT-A injection on the decrease of masseter muscle intensity is clear. Considering the destructive consequences of an extreme amount of bruxism force, BoNT-A injection can be a good modality to control the intensity of SB.

None of the sleep variables showed a significant time and group interaction. However, percentages of N2 and REM stages and sleep efficiency changed significantly in the placebo group during the 12 weeks. Total sleep time, percentage of N1 stage, and arousal index changed significantly in the treatment group during the 12 weeks. Subjects showed significant differences in some sleep variables for three PSG recordings. Our subjects showed lower sleep efficiency (mean 77–82%) than the normal range (80–85%) and more frequent arousal (mean arousal index: 32–41) than the normal range (arousal index < 10). We supposed that there was a first-night effect. However, the first-night effect had no effect on the severity of RMMA, because the RMMA episodes/h were not correlated to the above sleep variables. PSG in a sleep laboratory is an absolutely accurate method, but it is very inconvenient to subjects because of the many electrodes and lines attached and the change in sleeping environment, leading to the first-night effect. The first-night effect is abnormally poor sleep on the first night of PSG, including lower sleep efficiency, frequent arousal, shorter sleep time, and longer sleep latency, and it may even last more than one night [26,27]. A previous study showed no first-night effect on the severity of RMMA frequency in young and healthy patients with SB [28]. In other studies, home PSG study showed a first-night effect in patients with low SB activity (2–4 episode index and/or < 25 burst index), and the first-night effect appeared to be combined with high night-to-night variability of SB activity [29]. In an SB study, it is recommended to perform several consecutive nights of PSG study, especially for subjects with low first-night SB activity to minimize the first-night effect and night-to-night variability of SB. This will help to confirm the real level of ongoing SB activity. One-night sleep recording may be sufficient for moderate to high frequency SB patients [28,29].

There are various treatment modalities for the management of SB, such as an occlusal splint, biofeedback, behavioral approaches, and pharmacological management (botulinum toxin, clonidine, clonazepam, etc.). Presently, there is not enough evidence to define a standard of reference approach for SB treatment, except for the use of occlusal splint [30]. Depending on where the focus of the SB treatment is, the choice of treatment will vary at the individual level. In this regard, botulinum toxin injection can also be a good modality.

There are several limitations of this study. First, the sample size (*n* = 23) was relatively small. Second, we evaluated the effect of BoNT-A for SB focused on efficacy and objective PSG findings. Subjective improvement is also important in clinical use. In the extending study, the subjective effectiveness of BoNT-A for SB should be evaluated. Third, we selected subjects among occlusal splint users for SB. They showed moderate to severe wear facets on their splint. We supposed that they were moderate to high frequency SB patients. However, some showed low frequency of SB due to night-to-night variability or first night effect of PSG. Due to budgetary limitations, we did not perform several consecutive nights of PSG study. Finally, subjects showed lower sleep efficiency and significant differences in some sleep variables for three PSG recordings. One of the reasons for these results might be the first-night effect of PSG. Several consecutive nights of PSG study should be performed to improve these problems.

## 4. Conclusions

This study is significant for evaluating the long-term effect of BoNT-A for SB using PSG evaluation in a randomized, placebo-controlled trial. We confirmed that BoNT-A cannot control the genesis of RMMA. Rather, it might be a management option for controlling masticatory muscle intensity during SB and protecting the orofacial structures from the excessive forces. Changing the concept of SB, i.e., from disorder to behavior, we can use BoNT-A as an effective modality in reducing the intensity of masticatory muscle during SB along with occlusal splints. In the future, we need randomized, double-blind, placebo-controlled clinical studies with an accurate SB diagnosis by several consecutive PSG recordings and large sample size.

## 5. Methods and Materials

### 5.1. Standard Protocol Approvals, Registrations, and Patient Consents

The protocol of this study was undertaken with the approval of Institutional Review Board (IRB) of the Yonsei University Dental Hospital (IRB number: 2-2016-0016, approval date: 7 July 2016). This trial was registered on the Clinical Research Information Service (CRiS) of Ministry of Health and Welfare of Republic of Korea (CRiS number: KCT 0004459). All participants were informed of the nature of the study, and written consent was obtained from each participant.

### 5.2. Study Design and Subjects

Thirty SB subjects (11 males, 19 females; ages 20 to 56 years) were selected from outpatients at the Department of Orofacial Pain and Oral Medicine, Yonsei University Dental Hospital. All subjects were interviewed by one clinician (S.T.K.), who documented the subject’s age, gender, medical history, and drug history at the first visit. The subjects had the following clinical signs and symptoms for SB: (1) a history of tooth grinding occurring at least three nights per week; (2) experience of morning jaw stiffness; and (3) clinical presence of tooth wear [1,31]. They had been using an occlusal splint for SB. However, they still had self-reported bruxism activity and showed moderate to severe wear facets on their occlusal splint. The following exclusion criteria were applied: (1) previously received BoNT-A injection into both the masseter and the temporalis muscles for the past one year; (2) taking medications affecting muscle relaxation (e.g., antiepileptic drugs, benzodiazepines, aminoglycoside, curare-like agents); (3) infectious skin lesion at the site of injection; (4) allergy to BoNT-A; (5) neuromuscular disease; and (6) pregnant females.

This study was a randomized, double-blind, placebo-controlled, 1:1, parallel-design trial. The subjects were randomly assigned into two groups. The placebo group (15 subjects) received saline injections into the bilateral masseter muscles, and the treatment group (15 subjects) received BoNT-A injections into the bilateral masseter muscles. The subjects were requested not to take any other treatments or medications that would affect the muscles and not to use an occlusal splint during the period of this study. The subjects were blinded as to which group they were in.

### 5.3. Botulinum Toxin

The BoNT-A (prabotulinumtoxin A; Daewoong Pharmaceutical, Seoul, Korea) was supplied as a freeze-dried powder of 100 U and was reconstituted with 2 mL of sterile normal saline to a concentration of 5 U/0.1 mL. A dose of 25 U of BoNT-A was injected into each masseter muscle using a 1-mL syringe with a 29-gauge, 0.5-inch needle. BoNT-A was injected into two sites of each subject’s masseter muscle in the treatment group, referring to previous studies [32,33]. Injection sites were separated by 1–2 cm in the center of the middle third of the masseter muscle, in which the first site was the inferior, most prominent part of the masseter muscle when the subject was asked to clench, and the other site was 1–2 cm vertically away from the first site. In the placebo group, instead of BoNT-A, the same amount of normal saline was injected into two sites.

### 5.4. Audio–Video–Polysomnography (PSG) in the Sleep Laboratory

All subjects were studied in the sleep laboratory for three nights. The first night was before injection, the second night was 4 weeks after injection, and the last night was 12 weeks after injection. We used the same PSG recording methodology as the one employed in our previous study [15].

Prior to sleep recordings, each patient completed a series of oromotor tasks to enable signal recognition and calibration of EMG amplification. Biocalibration for the masseter muscle EMG recordings included maximal voluntary clencing (MVC) and lateral jaw movement. The sleep recordings commenced at 22:30 (± 30 min) and ended upon the subject’s spontaneous awakening or at 06:00. PSG montage included electrooculography, electroencephalography (EEG), electrocardiography, abdominal and chest bands, pulse oximetry, and EMG recordings from the submental and bilateral tibialis anterior muscles, as well as from the bilateral masseter muscles. Video (focused on the head and neck area) and audio recordings were made simultaneously. All signals were amplified and recorded at a sampling rate of 200 Hz and stored for off-line analysis using Embla RemLogic 2.0 software (Natus, Middleton, WI, USA).

### 5.5. Data Analysis

All anonymized recordings from the 30 subjects were analyzed with RemLogic 2.0 software (Natus, Middleton, WI, USA) in randomized order. A single observer (H.C.H.) scored sleep variables and the other single observer (S.Y.J.) scored RMMA events.

#### 5.5.1. Scoring of Sleep Variables

Sleep stages and microstructures, apnea and hypopnea events, and periodic leg movements in sleep were scored according to the diagnostic and coding manual of American Academy of Sleep Medicine [1]. The following sleep parameters were calculated: total sleep time, sleep stage, sleep efficiency, sleep latency, arousal index, apnea–hypopnea index, and periodic limb movement index.

#### 5.5.2. Scoring of Sleep Bruxism Episodes

RMMA, a characteristic EMG pattern of the masseter muscle during sleep, was scored to make a polysomnographic diagnosis of SB. There are three types of SB episodes: phasic (three EMG bursts with durations of 0.25–2.0 s), isolated tonic bursts (EMG bursts lasting > 2.0 s), and mixed types (both phasic and tonic bursts) [34]. Episodes were scored as a single episode when they were separated by intervals of less than 3 s. We used the same SB episode scoring methodology as the one employed in a previous study [15]. For SB episodes, the number of RMMA episodes per hour of sleep (RMMA episodes/h), bursts per hour of sleep (bursts/h), the number of bursts per episode (bursts/episode), the mean episode duration, and the number of episodes with sound (episodes sound) were calculated. In addition, the peak amplitude of an EMG burst of the masseter muscles during SB episode was measured by ruler on the screen, and maximum peak value was calculated for each episode.

### 5.6. Statistical Analysis

The normality of the data distribution was verified by the Shapiro–Wilk test. The data that were not normally distributed underwent logarithmic transformation. A two-sample *t*-test and a paired *t*-test were performed when the interaction between time and group was significant. RM-ANOVA were used to find out whether differences existed between the placebo and treatment groups. The Fisher’s exact test was used to compare the differences according to sex. Statistical analysis was performed using the IBM SPSS version 23.0 (IBM Corp., Armonk, NY, USA) at a significance level of *p* < 0.05.

## Figures and Tables

**Table 1:** Characteristics of the subjects.

Variables	Placebo (*n* = 10)	Treatment (*n* = 13)	*p* Value
**Age,** years ^a^	28.90 ± 8.13	32.46 ± 9.94	0.19
**Sex** (*n*) ^b^	F: 6, M: 4	F: 7, M: 6	1.00
**BMI,** kg/m^2 c^	21.39 ± 3.22	22.31 ± 2.80	0.47

BMI, body mass index. ^a^ Data are analyzed by Wilcoxon rank sum test. ^b^ Data are analyzed by Fisher’s exact test. ^c^ Data are analyzed by two-sample *t*-test.

**Table 2:** Sleep variables.

Sleep Variables	Baseline (Mean ± SD)	4 Weeks (Mean ± SD)	12 Weeks (Mean ± SD)	Time ^†^ (*p* Value)	Interaction * (*p* Value)
TST (min)				0.154	0.089
Placebo	307.10 ± 39.90	296.35 ± 52.06	332.29 ± 29.57	0.078	
Treatment	320.46 ± 44.24	290.27 ± 35.60	293.08 ± 65.95	0.009	
Stage N1 (%)				0.102	0.567
Placebo	8.66 ± 4.49	11.06 ± 6.54	10.37 ± 8.66	0.687	
Treatment	9.27 ± 2.99	12.73 ± 5.24	14.31 ± 8.78	0.040	
Stage N2 (%)				0.069	0.487
Placebo	51.84 ± 6.45	48.85 ± 10.36	45.38 ± 8.48	0.008	
Treatment	51.21 ± 8.86	51.37 ± 7.59	49.09 ± 8.64	0.564	
Stage N3 (%)				0.694	0.286
Placebo	17.93 ± 5.38	21.89 ± 8.08	18.36 ± 8.97	0.375	
Treatment	19.51 ± 8.80	17.26 ± 10.29	17.45 ± 7.94	0.616	
Stage REM (%)				0.077	0.130
Placebo	21.57 ± 6.95	17.18 ± 9.19	25.89 ± 8.45	0.040	
Treatment	20.02 ± 7.61	18.63 ± 7.62	19.15 ± 9.60	0.860	
Sleep efficiency (%)				0.150	0.177
Placebo	81.46 ± 9.42	76.83 ± 11.06	87.43 ± 6.66	0.047	
Treatment	83.08 ± 9.27	78.17 ± 8.92	78.89 ± 17.58	0.080	
Sleep latency (min)				0.442	0.496
Placebo	21.63 ± 21.46	11.48 ± 7.65	12.65 ± 7.38	0.411	
Treatment	15.94 ± 15.46	16.58 ± 12.72	13.9 ± 25.85	0.908	
Arousal index				0.028	0.763
Placebo	29.80 ± 15.80	39.08 ± 16.28	30.37 ± 15.78	0.133	
Treatment	35.23 ± 13.02	43.71 ± 11.99	39.54 ± 16.83	0.008	
AHI				0.310	0.467
Placebo	1.60 ± 1.95	1.86 ± 2.44	1.63 ± 3.24	0.811	
Treatment	12.89 ± 21.24	14.78 ± 23.13	10.72 ± 17.01	0.310	
PLM index				0.809	0.251
Placebo	2.24 ± 3.82	1.31 ± 2.02	1.85 ± 4.24	0.749	
Treatment	0.42 ± 0.91	2.34 ± 4.86	1.42 ± 3.17	0.280	

Abbreviations: TST, total sleep time; AHI, apnea–hypopnea index; PLM, periodic limb movement. Data are presented as mean ± standard deviation values and underwent log transformed for comparison. ^†^ Intragroup and * intergroup interactions were evaluated by repeated measures ANOVA.

**Table 3:** Sleep bruxism variables.

Sleep Bruxism Variables	Baseline (Mean ± SD)	4 Weeks (Mean ± SD)	12 Weeks (Mean ± SD)	Time ^†^ (*p* Value)	Interaction * (*p* Value)
**RMMA episode variables**					
Episodes/h				0.055	0.243
Placebo	3.26 ± 3.98	4.62 ± 3.52	2.33 ± 2.47	0.036	
Treatment	5.08 ± 4.20	4.81 ± 2.94	5.15 ± 5.06	0.813	
Bursts/h				0.291	0.261
Placebo	18.99 ± 26.60	25.34 ± 24.22	11.95 ± 14.59	0.110	
Treatment	40.72 ± 67.33	31.90 ± 38.42	45.63 ± 73.18	0.711	
Bursts/episode				0.609	0.780
Placebo	4.41 ± 1.65	4.36 ± 2.45	4.59 ± 1.30	0.435	
Treatment	6.01 ± 4.93	5.55 ± 3.04	6.24 ± 4.13	0.796	
Episode duration (s)				0.863	0.708
Placebo	5.25 ± 2.91	5.38 ± 2.88	5.20 ± 1.65	0.850	
Treatment	7.10 ± 5.45	5.54 ± 2.73	6.17 ± 3.93	0.715	
Episodes sound				0.563	0.137
Placebo	8.10 ± 17.78	9.30 ± 17.44	4.30 ± 12.22	0.490	
Treatment	16.69 ± 20.89	8.46 ± 15.25	18.15 ± 26.01	0.162	
**EMG variables**					
MVC MA (μV)				< 0.0001	0.044
Placebo	118.00 ± 94.73	106.50 ± 105.94	92.00 ± 109.83	0.193	
Treatment	89.23 ± 55.86	38.08 ± 20.57	36.69 ± 24.44	<0.0001	
RMMA MA (μV)				< 0.0001	0.001
Placebo	71.46 ± 46.94	62.82 ± 32.98	61.42 ± 50.90	0.356	
Treatment	88.05 ± 70.25	29. 80 ± 13.94	37.24 ± 23.80	< 0.0001	

Abbreviations: RMMA, rhythmic masticatory muscle activity; EMG, electromyography; MVC MA, the peak amplitude of EMG burst in the masseter muscle during maximal voluntary clenching; RMMA MA, the peak amplitude of EMG burst in the masseter muscle during rhythmic masticatory muscle activity. Data are presented as mean ± standard deviation values and underwent log transformation for comparison. ^†^ Intragroup and * intergroup interactions were evaluated by repeated measures ANOVA.
